# Supplementary material for: Estimating dengue transmission intensity from serological data: A comparative analysis using mixture and catalytic models
Source: PLoS Negl Trop Dis. 2022 Jul 11;16(7):e0010592. doi: 10.1371/journal.pntd.0010592 (PMC9302823; doi:10.1371/journal.pntd.0010592)
Supplement: S1 Table — (DOCX) [file pntd.0010592.s001.docx]

**S1 Table: Parameter values used for generating 540 simulated datasets.**

| Parameter | Symbol | Uniform distribution limits |
| --- | --- | --- |
| Force of infection (FOI) | $\lambda$ | 0.001 – 0.180 |
| Mean seronegative log(titre+1) | $\mu_{S}$ | 0.05 – 2.00 |
| Mean seropositive log(titre+1) | $\mu_{I}$ | 2.0 – 4.0 |
| Standard deviation of the seronegative log(titre+1) distribution | $\sigma_{S}$ | 0.1 – 1.0 |
| Standard deviation of the seropositive log(titre+1) distribution | $\sigma_{I}$ | 0.1 – 1.0 |
