# Supplementary material for: Estimating dengue transmission intensity from serological data: A comparative analysis using mixture and catalytic models
Source: PLoS Negl Trop Dis. 2022 Jul 11;16(7):e0010592. doi: 10.1371/journal.pntd.0010592 (PMC9302823; doi:10.1371/journal.pntd.0010592)
Supplement: S2 Table — Here, n represents the number of simulated datasets out of 540. (DOCX) [file pntd.0010592.s002.docx]

**S2 Table:** **Number of simulations where the mixture model correctly specified the distributions of the seronegative and/or seropositive component of the simulated antibody titre datasets (Dataset C).** Here, n represents the number of simulated datasets out of 540.

| True seronegative distribution | True seropositive distribution | n | Correct seronegative distribution only | Correct seropositive distribution only | Correct both distributions | Incorrect both distributions |
| --- | --- | --- | --- | --- | --- | --- |
| Weibull | **Weibull** | **60** | 6 | 5 | 46 | 3 |
| Weibull | **Normal** | **60** | 13 | 5 | 40 | 2 |
| Weibull | **Gamma** | **60** | 5 | 7 | 48 | 0 |
| Normal | **Weibull** | **60** | 16 | 5 | 37 | 2 |
| Normal | **Normal** | **60** | 13 | 0 | 47 | 0 |
| Normal | **Gamma** | **60** | 11 | 2 | 46 | 1 |
| Gamma | **Weibull** | **60** | 1 | 5 | 53 | 1 |
| Gamma | **Normal** | **60** | 6 | 8 | 46 | 0 |
| Gamma | **Gamma** | **60** | 8 | 4 | 48 | 0 |
