# Supplementary material for: Estimating dengue transmission intensity from serological data: A comparative analysis using mixture and catalytic models
Source: PLoS Negl Trop Dis. 2022 Jul 11;16(7):e0010592. doi: 10.1371/journal.pntd.0010592 (PMC9302823; doi:10.1371/journal.pntd.0010592)
Supplement: S3 Table — The observed data is serology data collected in Vietnam (Datasets A-1:A-6) and Indonesia (Dataset B). 95% Confidence Intervals (CI) were calculated by the bootstrap method. (DOCX) [file pntd.0010592.s003.docx]

**S3 Table: Force of infection (FOI) and total population level seroprevalence (SP) estimates from the mixture model and the catalytic models fitted to the observed data.** The observed data is serology data collected in Vietnam (Datasets A-1:A-6) and Indonesia (Dataset B). 95% Confidence Intervals (CI) were calculated by the bootstrap method.

| Dataset | Parameter | Mixture model  (95% CI) | Time-varying catalytic model (95% CI) | Time-constant catalytic model (95% CI) |
| --- | --- | --- | --- | --- |
| A-1 | FOI | 0.026 (0.019-0.033) | 0.034 (0.011-0.076) | 0.031 (0.028-0.034) |
|  | SP | 0.163 (0.138-0.188) | 0.203 (0.173-0.234) | 0.203 (0.186-0.219) |
| A-2 | FOI | 0.099 (0.077-0.124) | 0.050 (0.012-0.118) | 0.037 (0.035-0.039) |
|  | SP | 0.322 (0.293-0.352) | 0.278 (0.244-0.312) | 0.280 (0.266-0.294) |
| A-3 | FOI | 0.085 (0.069-0.102) | 0.037 (0.006-0.098) | 0.033 (0.031-0.036) |
|  | SP | 0.376 (0.349-0.403) | 0.262 (0.229-0.297) | 0.264 (0.250-0.278) |
| A-4 | FOI | 0.039 (0.017-0.069) | 0.024 (0.007-0.058) | 0.023 (0.021-0.024) |
|  | SP | 0.265 (0.232-0.296) | 0.189 (0.163-0.217) | 0.190 (0.178-0.202) |
| A-5 | FOI | 0.073 (0.056-0.091) | 0.045 (0.005-0.102) | 0.030 (0.028-0.032) |
|  | SP | 0.301 (0.273-0.330) | 0.253 (0.217-0.291) | 0.255 (0.240-0.270) |
| A-6 | FOI | 0.034 (0.022-0.050) | 0.038 (0.007-0.101) | 0.034 (0.031-0.036) |
|  | SP | 0.254 (0.225-0.283) | 0.299 (0.262-0.337) | 0.300 (0.282-0.318) |
| B | FOI | 0.154 (0.106-0.213) | 0.164 (0.022-0.814) | 0.143 (0.136-0.150) |
|  | SP | 0.718 (0.694-0.741) | 0.700 (0.655-0.743) | 0.700 (0.686-0.714) |
