# Supplementary figures and images for: Estimating dengue transmission intensity from serological data: A comparative analysis using mixture and catalytic models
Source: PLoS Negl Trop Dis. 2022 Jul 11;16(7):e0010592. doi: 10.1371/journal.pntd.0010592 (PMC9302823; doi:10.1371/journal.pntd.0010592)

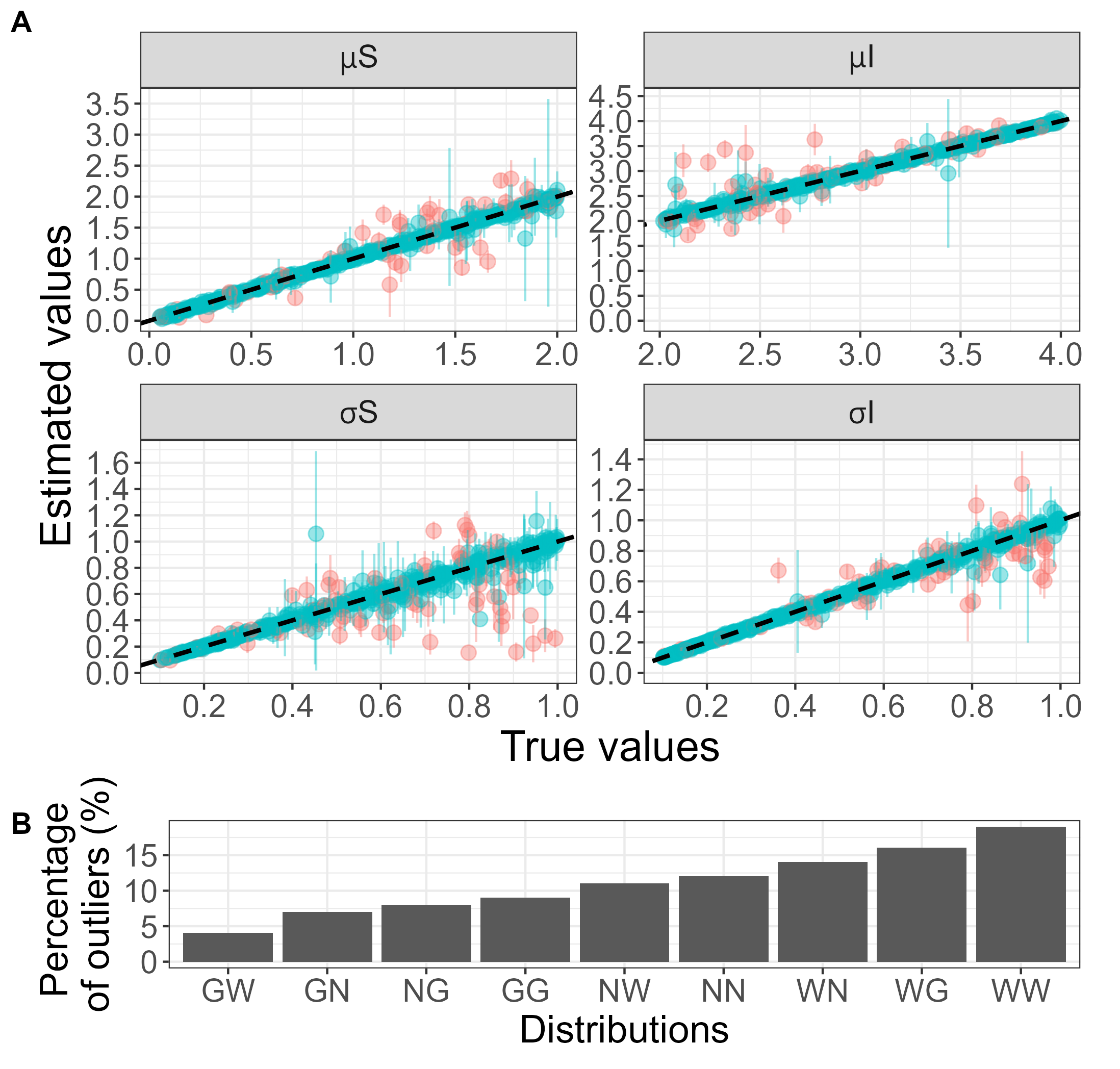

Supplement: S1 Fig — (A) True versus estimated parameter values from the mixture model fitted to the simulated datasets (Dataset C). The estimated parameters are the mean log(titre + 1) value of the seronegative/susceptible (S) and seropositive/infected (I) components (μs and μI respectively) and the corresponding standard deviations (σs and σI). Red indicates the estimates where the true parameter value was not captured by the estimates (i.e., the 95% Confidence Interval of the estimate did not contain the true value). Note that the axes limits differ for each panel. (B) The percentage of parameter outliers after fitting the mixture model to Dataset C, per seronegative and seropositive titre family distributions. The percentage of the total number of outliers of μs, μI, σS and σI (red in panel A) per distribution combination on the x-axis, where the two letters represent the seronegative (first letter) and the seropositive (second letter) distribution pair (N = normal, G = gamma and W = Weibull). (TIF) [file pntd.0010592.s004.tif]

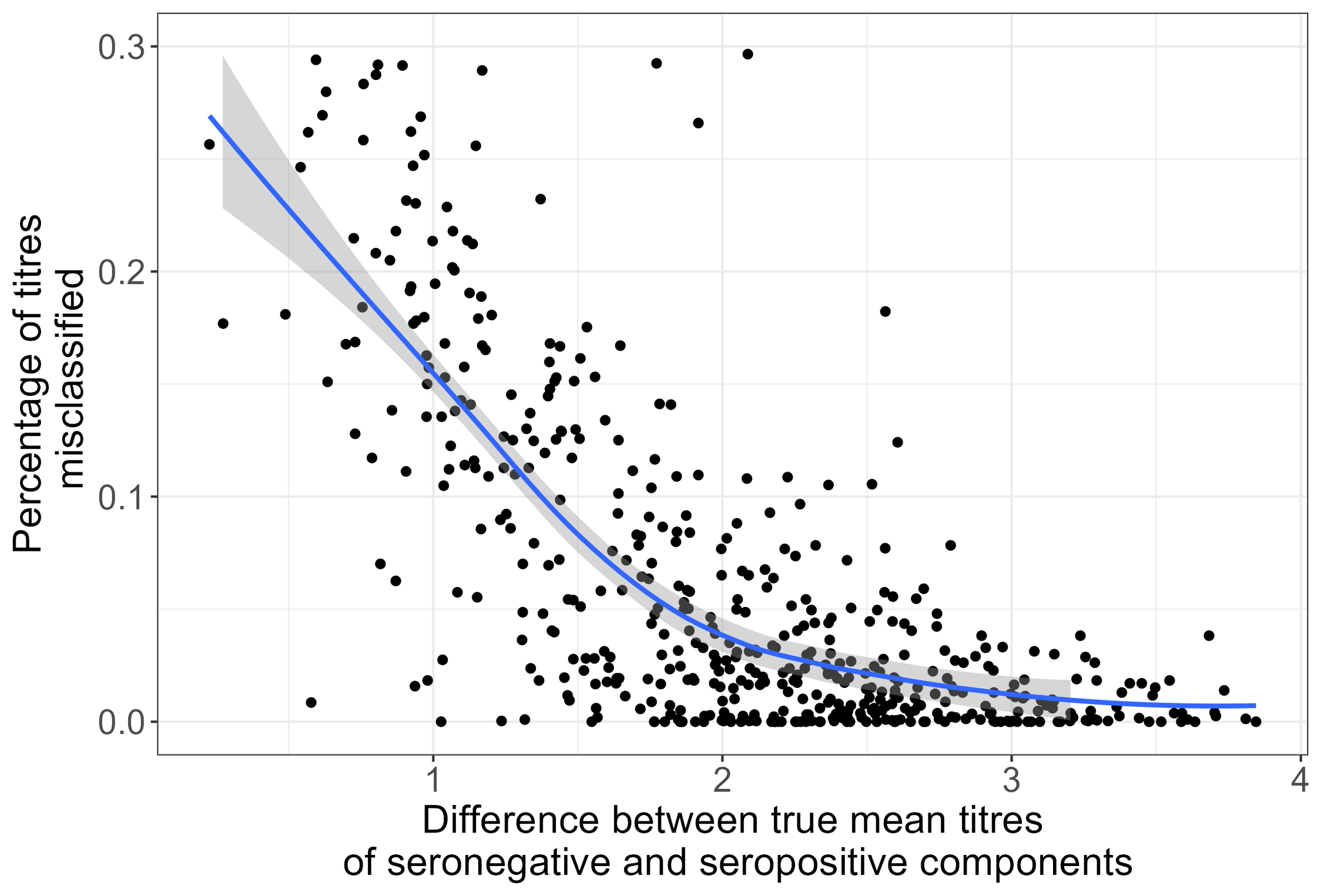

Supplement: S2 Fig — The x-axis shows the difference between the true mean log(titre + 1) value of the seronegative (μs) and the seropositive component (μI) for each realisation over 509 simulated datasets. The titres are classified as seropositive or seronegative using realisation-specific optimised titre thresholds. The loess regression line and corresponding 95% Confidence Intervals are shown. (TIF) [file pntd.0010592.s005.tif]

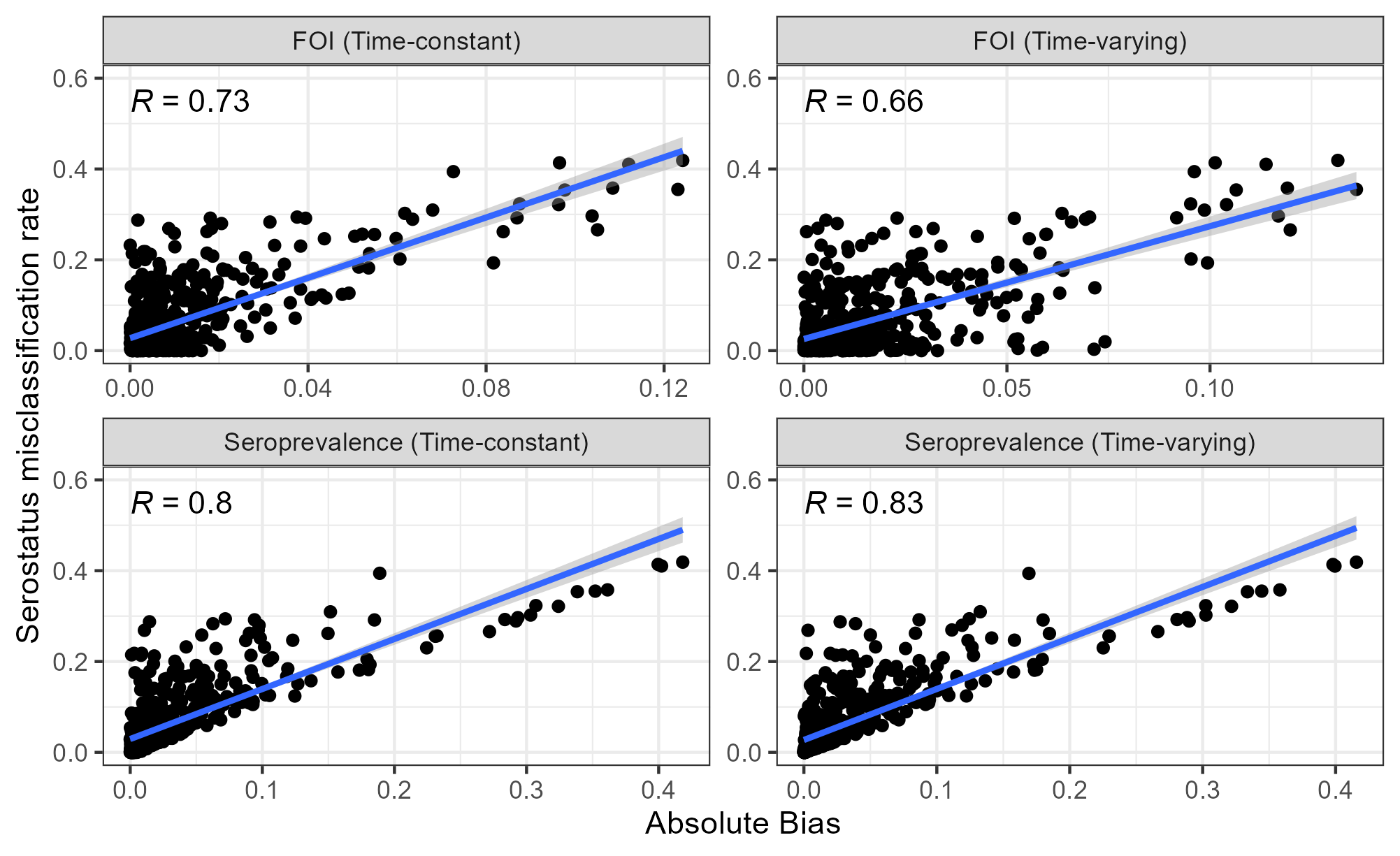

Supplement: S3 Fig — The bias in the estimates from the time-constant and time-varying catalytic models for the realisations over 509 simulated datasets are plotted against the serostatus misclassification error rate. The serostatuses of the titres in each of the 509 simulated datasets are classified as seropositive or seronegative using realisation-specific optimised titre thresholds. The serostatus misclassification error rate is calculated as the percentage of titres in each dataset that are misclassified. Absolute bias is calculated as the absolute value of the estimated value–true value for the force of infection (FOI) and seroprevalence. The linear regression lines and corresponding 95% Confidence Intervals are shown, as well as the Pearson’s correlation coefficients (R). Three outliers with FOI estimates > 0.4 were removed from the time-varying catalytic model panel and corresponding regression line estimation. (TIF) [file pntd.0010592.s006.tif]

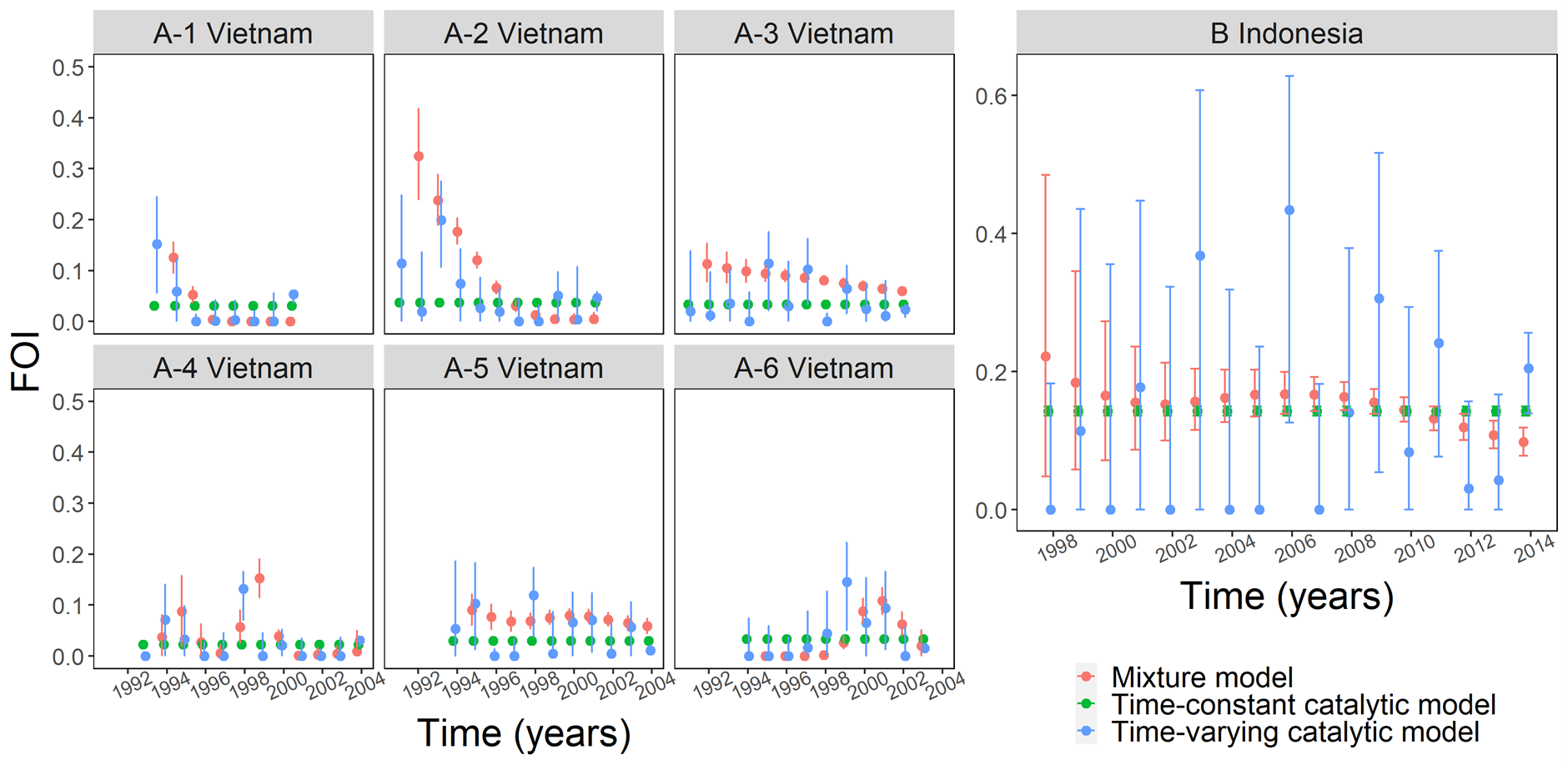

Supplement: S4 Fig — Yearly FOI estimates from the catalytic and mixture models. 95% Confidence Intervals were calculated by bootstrapping. (TIF) [file pntd.0010592.s007.tif]
