# Supplementary material for: Estimating dengue transmission intensity from serological data: A comparative analysis using mixture and catalytic models
Source: PLoS Negl Trop Dis. 2022 Jul 11;16(7):e0010592. doi: 10.1371/journal.pntd.0010592 (PMC9302823; doi:10.1371/journal.pntd.0010592)
Supplement: Attachment — Submitted filename: PNTD-D-21-01566 comments LC.docx [file pntd.0010592.s008.docx]

General comments:

The authors describe an analysis of field data on dengue antibody titres, using three different models. In a simulation exercise, they first quantify and qualify the behaviour of the three models, assessing bias, uncertainty, and coverage. They then proceed to fit the models to two datasets from Vietnam (multiple years) and Indonesia (one year). Although I intuitively appreciate the relevance of the work, several methodological details are missing or described in an inefficient way, making it hard to assess the appropriateness of the approach. In addition, several sections of supplemental information seem to be missing from the package supplied to the reviewers.

**Abstract**

Lines 28-32 in the results section of the abstract report a greater positive FOI bias (overestimation) for catalytic models, based on simulated data. However, when analysing the results of the real field data, the mixture models produced higher FOI estimates. This is somewhat counterintuitive; was the bias reported in lines 28-32 perhaps a negative bias (underestimation)? After reading the entire paper, it becomes (somewhat) clearer what the reported biases represent and that at some point the biases are converted to absolute values. See more comments on this below.

**Methods**

Lines 142-143 state that equivocal titres in the Indonesian data were discarded. However, as the authors mention in the introduction, these can be included and analysed with the mixture model. Therefore, if possibly, these data should be included in the mixture model analysis, as they are part of the “benefits” of using a mixture model.

If I understand correctly, the time-constant catalytic model was fitted to each survey separately. However, it’s not clear to me how the time-varying catalytic model works; I would expect such a model to be fitted simultaneously to all the Vietnamese surveys for all years, such that the FOI for age *a* at time *t* also factors into the seroprevalence for age *a*+1 at time *t*+1. But from the figures, I deduce that the time-varying catalytic model was also fitted to single surveys (e.g., the Indonesian data), and I don’t understand how a single cross-sectional survey can provide information on both (1) cohort effects (age-dependent exposure) and (2) period effects (time-dependent exposure). Did the authors perhaps actually fit “age-constant” and “age-varying” catalytic models, rather than “time-constant” and “time-varying”? This may perhaps become clearer when more carefully explained in the methods section.

Please clarify what the normal, gamma and Weibull distribution represent in the simulation analysis. The distribution of titres, log-titres, or log(titres + 1)? Also, shouldn’t “normal” be “log-normal”, such that the support (i.e., positive real numbers) is the same for the three distributions?

When fitting the mixture model, the best-fitting combination of distributions was based on the log-likelihood ratio statistic. However, this approach assumes that models are nested, which the models based on different combinations of distributions are not. Instead, an information criterion (i.e., the LR plus the estimated effective number of parameters in the model, which I imagine may not necessarily be the same between the models) should be used to compare the models based on the different combinations of distributions.

Also, again please clarify what the normal, gamma, and Weibull distributions pertain to (titres, log-titres, or log(titres + 1)) within the mixture model.

Line 199-200: why did the authors consider to fix the standard deviations of the two mixture distributions? Couldn’t these be estimated from the data?

I’m not sure but I wonder how appropriate it is to base estimates of mu’(a) on the gradient of the spline at a specific age, as I imagine the curvature of the spline can be quite variable around age a. Also, was it strictly necessary to use splines, which are prone to overfitting data? Did the authors consider more structure approaches to quantify the age pattern and assess the performance of such approaches?

Although not specified anywhere, I take it that all of the models used in this study assume that there is no seroreversion? Is this reasonable for dengue? (I don’t know as I’m not a dengue expert.)

The choice of uniform distributions for the input parameters of the simulation exercise is counterintuitive. Ideally, these distributions represent a reasonable expectation about values that occur in “reality”. Such an expectation would be much better represented by a distribution with tails. Currently, more “extreme” values for input parameters are given equal importance in the analysis as “more reasonable” values towards the middle of a distribution. I cannot oversee the potential consequences of these choices, but they might affect the differences between the models in terms of performance.

**Results**

The results of the simulation-based bias analysis are presented in terms of absolute bias, which obscures whether titres/seroprevalences were (or were not) generally over- or underestimated. Also, the methods section should already explain how bias was calculated and assessed. This is now explained for the first time in the results section (lines 237-238), although there is even an inconsistency there. The statement “Bias was calculated as the estimated parameter values minus the true parameter values” suggests that biases were assess on the FOI scale (which is mentioned in the previous sentence). However, the next sentence (lines 238-241) speaks of “absolute bias in the FOI estimates” in terms of %, which suggests a relative scale. Or is this an error and referring to percentage-points bias on the seroprevalence scale? This all needs more careful explanation and description.

Figure 1: it might be useful to indicate the target coverage (bottom right panel), which I assume is 95%?

Table S3 reports “Percentage of simulations” that correctly specified seropos/neg components. Shouldn’t this be “the proportion of individuals correctly identified as seropos/neg, averaged over the simulations”? I also noticed that the number of simulations differs for each combination of distributions for seropos/neg titres, which means that the Monte Carlo error of the estimates reported in the tables differs for each combination of distributions. Ideally, perform the stated 200 simulations for each combination. Also, as little as 12 simulations (for the normal-normal distribution combination) is too few to base inferences on. Statements like lines 225-227 in the main text (about the overrepresentation of Weibull-distributed seronegative titres among misidentified distributions) should be based on more simulations, and ideally the same number of simulations per combination of distributions.

Figure S1 summarises the estimated vs. true parameter values in the simulation study. Could the authors perhaps checks, and if relevant, visualise whether specific combinations of distributions where overrepresented among the outlying estimates (red bullets)?

Figure S2: in the figure legend, clarify what this “%” misclassification error represents. Also, the use of linear regression to summarise the pattern is inappropriate as the “data” are bounded at zero (and the regression line dives below zero within the range of the “data”).

Figure S3: looking purely at the “data”, I would say there is no or little correlation between the two outcomes on the x and y axis. In contrast, the linear regression lines are very suggestive of a strong correlation, however, these are largely driven by very few data points towards the right end of the x axis.

Lines 253-255 refer to S7 Figure, but no such figure is included in the supplementary materials. Several other cited supplementary materials are not available to the reviewer.

Lines 255-258: I’m not sure I follow why the models were fitted to the “true serostatus”, or how this was done.

Reading about the existence of multiple districts in the Vietnam data, I don’t recall reading anything about it in the methods section and whether and how this was dealt with in the modelling.

**Discussion**

For a (too) large part, the discussion section rehashes the results, with many references to tables and figures. For instance, the third paragraph starts by listing of literal estimates. As a reader, I’m left wondering what it all means. I was expecting to get at least some sense of this by the end of the first paragraph in the discussion (and then getting more details later). A more high-level description of the findings and implications would be helpful here.

I am missing a link to the motivation for the (a) motivation for the study as phrased in the author summary: “Characterising the transmission intensity of dengue virus in different geographic areas over time is essential to understand who is at greatest risk of infection, and to inform the implementation of interventions, such as vector control and vaccination.” If FOI estimates are important to understand where dengue transmission is most intense and to inform the implementation of interventions, bias in FOI estimates is only relevant if they muddle the relative ranking of different areas or (subsets of) populations in terms of transmission intensity and/or recency. This is not clearly discussed and I’m not sure whether the analyses and results are suitable to make statements about this.

**Minor comments**

- Line 52: semicolon should be a comma
- The order of the figures was mixed up in the pdf file for the reviewers, which caused some confusion at first.
