# Supplementary material for: Estimating dengue transmission intensity from serological data: A comparative analysis using mixture and catalytic models
Source: PLoS Negl Trop Dis. 2022 Jul 11;16(7):e0010592. doi: 10.1371/journal.pntd.0010592 (PMC9302823; doi:10.1371/journal.pntd.0010592)
Supplement: Attachment — Submitted filename: Reviewer_feedback_response_letter.docx [file pntd.0010592.s009.docx]

**Point-by-point response letter**

**Estimating dengue transmission intensity from serological data: a comparative analysis using mixture and catalytic models**

Victoria Cox, Megan O’Driscoll, Natsuko Imai, Ari Prayitno, Sri Rezeki Hadinegoro, Anne-Frieda Taurel, Laurent Coudeville, Ilaria Dorigatti

# Reviewer #1

**General comments:**

The authors describe an analysis of field data on dengue antibody titres, using three different models. In a simulation exercise, they first quantify and qualify the behaviour of the three models, assessing bias, uncertainty, and coverage. They then proceed to fit the models to two datasets from Vietnam (multiple years) and Indonesia (one year). Although I intuitively appreciate the relevance of the work, several methodological details are missing or described in an inefficient way, making it hard to assess the appropriateness of the approach.

Thank you for your feedback and for bringing to our attention that the methods were not clear enough. We have now incorporated a more complete description of the methods in the main text including all details and steps implemented to generate the results. We believe that the revised manuscript is greatly improved and describes the methods with more clarity and efficiency.

In addition, several sections of supplemental information seem to be missing from the package supplied to the reviewers.

Thank you for bringing this to our attention. Unfortunately, there was a labelling issue in the Supplementary Information that caused the confusion, which has now been rectified. Apologies for the confusion that this has caused.

**Abstract**

Lines 28-32 in the results section of the abstract report a greater positive FOI bias (overestimation) for catalytic models, based on simulated data. However, when analysing the results of the real field data, the mixture models produced higher FOI estimates. This is somewhat counterintuitive; was the bias reported in lines 28-32 perhaps a negative bias (underestimation)? After reading the entire paper, it becomes (somewhat) clearer what the reported biases represent and that at some point the biases are converted to absolute values. See more comments on this below.

Yes, the values included in the original abstract were absolute bias estimates. We have changed this to give the actual bias rather than its absolute value. Thank you for the suggestion, we hope this makes interpreting the results clearer.

Summary of changes:

- Lines 28-31 now read: “The simulation study showed larger mean FOI estimate bias from the time-constant and time-varying catalytic models (-0.007 (95% Confidence Interval (CI): -0.069, 0.029) and -0.006 (95% CI -0.095, 0.043)) than from the mixture model (0.001 (95% CI -0.036, 0.065)).”

**Methods**

Lines 142-143 state that equivocal titres in the Indonesian data were discarded. However, as the authors mention in the introduction, these can be included and analysed with the mixture model. Therefore, if possibly, these data should be included in the mixture model analysis, as they are part of the “benefits” of using a mixture model.

Thank you for bringing to our attention that this point was unclear. We confirm that the equivocal titres were included in the mixture model analysis, and this was one of the key reasons we wanted to explore this dataset using mixture models. The text has been updated to clarify that equivocal titres were included in the mixture model analysis but were discarded for the catalytic model analysis.

Summary of changes:

- Lines 181-185 in the mixture model methods section now read: “Mixture models were applied to the bimodal distribution of individual antibody titres as described in Bollaerts *et al*., 2012 and Hens *et al*., 2012. All individual antibody titre measurements were used in each dataset, which differs from the data used for the catalytic model where equivocal titres were discarded and titre measurements were classified as either seropositive or seronegative.”

If I understand correctly, the time-constant catalytic model was fitted to each survey separately. However, it’s not clear to me how the time-varying catalytic model works; I would expect such a model to be fitted simultaneously to all the Vietnamese surveys for all years, such that the FOI for age *a* at time *t* also factors into the seroprevalence for age *a*+1 at time *t*+1. But from the figures, I deduce that the time-varying catalytic model was also fitted to single surveys (e.g., the Indonesian data), and I don’t understand how a single cross-sectional survey can provide information on both (1) cohort effects (age-dependent exposure) and (2) period effects (time-dependent exposure). Did the authors perhaps actually fit “age-constant” and “age-varying” catalytic models, rather than “time-constant” and “time-varying”? This may perhaps become clearer when more carefully explained in the methods section.

Following Ferguson *et al*., 1999, we can use catalytic models fitted to single cross-sectional serosurveys to estimate FOI at age *a* years and *a+1* years, and interpret this as either the difference in the FOI experience by individuals at age *a* and age *a+1*, or the difference in the FOI experienced by all ages *a+1* years ago compared to *a* years ago. This is why in the original version of the manuscript we used the terms “time-varying” and “age-varying” interchangeably. However, we appreciate that this may cause confusion. Unfortunately, in the current modelling framework, it is not possible to disentangle time and age-specific heterogeneities in FOI. We have edited the methods section to clarify that in our ‘time-varying’ model we are estimating age-specific FOI.

Summary of changes:

- Lines 171 onwards read: “When we assumed a time-varying FOI, we estimated age-specific FOI 𝜆(𝑎𝑖) values, which were then averaged across the age groups (number of age groups = 𝑛) to produce a mean yearly FOI experienced over the study period (Equation 3). When we assumed a time-constant FOI, each 𝜆(𝑎𝑖) was assumed to equal 𝜆.”

Please clarify what the normal, gamma and Weibull distribution represent in the simulation analysis. The distribution of titres, log-titres, or log(titres + 1)? Also, shouldn’t “normal” be “log-normal”, such that the support (i.e., positive real numbers) is the same for the three distributions?

The distributions refer to log(titres + 1) within the mixture model. We simulated the data on the log scale which meant that the simulated values could exist in negative space. We therefore chose to include a normal distribution as one of the options to support the fitting of the mixture model to simulated data <0. The text has been edited to show this more clearly.

Summary of changes:

- Lines 129-132 now read: “For each simulation the distributions used for sampling seronegative and seropositive log(titres + 1) were selected from a normal, gamma or Weibull distribution. This gave 9 possible distribution pairs for seronegative and seropositive log(titre + 1) values, and we generated an equal number of simulations (N = 60) for each combination.”
- Lines 136-139 now read: “The serostatus of each individual was drawn from a Bernoulli distribution with probability 1−𝑒−λa, where 𝑎 is the age of the individual and λ is the FOI. FOI was assumed to be constant with age and time. Log(titre + 1) values for each individual were subsequently randomly drawn from the respective component distributions.”

When fitting the mixture model, the best-fitting combination of distributions was based on the log-likelihood ratio statistic. However, this approach assumes that models are nested, which the models based on different combinations of distributions are not. Instead, an information criterion (i.e., the LR plus the estimated effective number of parameters in the model, which I imagine may not necessarily be the same between the models) should be used to compare the models based on the different combinations of distributions.

Thank you for bringing this to our attention. We have re-run the mixture model on all the datasets and chosen the best-fitting mixture using the Akaike Information Criterion (AIC).

Summary of changes:

- Lines 193-197 read: “The *mixdist* R package was used to fit the mixture models to the titre data by maximum likelihood using an Expectation Maximisation (EM) algorithm (37). The package was adapted to allow fitting of different distributions for the seronegative and seropositive titre components: normal, gamma and Weibull distributions, giving 9 possible combinations. The best fitting mixture was chosen using the Akaike Information Criterion (AIC).”

Also, again please clarify what the normal, gamma, and Weibull distributions pertain to (titres, log-titres, or log(titres + 1)) within the mixture model.

The distributions refer to log(titres + 1) within the mixture model. The text has been edited to show this more clearly.

Line 199-200: why did the authors consider to fix the standard deviations of the two mixture distributions? Couldn’t these be estimated from the data?

When fitting the mixtures, if the standard deviations of the components were not fixed, the fitted seropositive component could overlap fully with the seronegative component, which is not biologically reasonable. Therefore, we chose to identify the best fitting mixtures, accounting for the biological context, by fixing the standard deviations of the mixture components to several different values and then choosing the best fitting mixture using the AIC.

I’m not sure but I wonder how appropriate it is to base estimates of mu’(a) on the gradient of the spline at a specific age, as I imagine the curvature of the spline can be quite variable around age a. Also, was it strictly necessary to use splines, which are prone to overfitting data? Did the authors consider more structure approaches to quantify the age pattern and assess the performance of such approaches?

We followed the methodology of Bollaerts *et al*., 2012 and Hens *et al*., 2010, and for this work we did not explore other approaches to quantify the age pattern. Because we used single 1-year age categories, we did not observe much variability in the curvature of the splines between each age point. It is a useful suggestion for future work to compare how and if different approaches to reconstruct mu(a) impact the final FOI estimates.

Although not specified anywhere, I take it that all of the models used in this study assume that there is no seroreversion? Is this reasonable for dengue? (I don’t know as I’m not a dengue expert.)

Correct, all the models assume that there is no seroreversion. We are using IgG antibody titres which are not serotype specific. Infection with one of the four serotypes of dengue provides lifelong immunity to homotypic infection (infection by the same serotype) and short-term immunity to heterotypic infection which does wane. While waning of heterotypic immunity could affect the IgG titres, this effect would be relevant only if a serosurvey was conducted soon after an epidemic. Because dengue is endemic in Vietnam and Indonesia it is fair to assume no seroreversion in the context of this analysis.

The choice of uniform distributions for the input parameters of the simulation exercise is counterintuitive. Ideally, these distributions represent a reasonable expectation about values that occur in “reality”. Such an expectation would be much better represented by a distribution with tails. Currently, more “extreme” values for input parameters are given equal importance in the analysis as “more reasonable” values towards the middle of a distribution. I cannot oversee the potential consequences of these choices, but they might affect the differences between the models in terms of performance.

We agree that in reality the distributions would have tails as opposed to being uniform. However, we deliberately chose to use a uniform distribution to fully explore the impact of using a wide range of (reasonable) parameter values, which better allows us to explore the effect of having overlapping seropositive and seronegative titre components. Applying our models to simulated datasets using uniform distributions for the parameters provides a more robust exploration of the methodology.

**Results**

The results of the simulation-based bias analysis are presented in terms of absolute bias, which obscures whether titres/seroprevalences were (or were not) generally over- or underestimated. Also, the methods section should already explain how bias was calculated and assessed. This is now explained for the first time in the results section (lines 237-238), although there is even an inconsistency there. The statement “Bias was calculated as the estimated parameter values minus the true parameter values” suggests that biases were assess on the FOI scale (which is mentioned in the previous sentence). However, the next sentence (lines 238-241) speaks of “absolute bias in the FOI estimates” in terms of %, which suggests a relative scale. Or is this an error and referring to percentage-points bias on the seroprevalence scale? This all needs more careful explanation and description.

Thank you for bringing this to our attention, we agree that the definition of bias needs to be included in the methods. Throughout the manuscript (except for Figure S6), we have changed the presentation of the figures and results to show bias, rather than the absolute value of the bias, to prevent obscuring over- and underestimation. In the original manuscript we presented the FOI and seroprevalence estimates as percentages, which meant that the bias in those estimates had a unit of % but was not a percentage bias. To ensure greater clarity, all FOI and seroprevalence results, and associated biases, have now been presented as decimal rather than percentages.

Summary of changes:

- Lines 231-235 in the methods section now read: “Bias in the mixture and catalytic model estimates of FOI and seroprevalence for Dataset C was calculated as the estimated value minus the true simulated value of the parameter. Uncertainty was calculated as the width of the 95% CIs around the parameter estimates. Coverage was calculated as the percentage of simulations where the estimated 95% CIs contained the true parameter value.”
- As an example of the format change for the presentation of FOI bias as decimals, the abstract results section now reads: “The simulation study showed larger mean FOI estimate bias from the time-constant and time-varying catalytic models (-0.007 (95% Confidence Interval (CI): -0.069, 29 0.029) and -0.006 (95% CI -0.095, 0.043)) than from the mixture model (0.001 (95% CI -0.036, 0.065)).”

Figure 1: it might be useful to indicate the target coverage (bottom right panel), which I assume is 95%?

Thank you for the suggestion, we have added a target coverage line to the panels.

Summary of changes:

Table S3 reports “Percentage of simulations” that correctly specified seropos/neg components. Shouldn’t this be “the proportion of individuals correctly identified as seropos/neg, averaged over the simulations”? I also noticed that the number of simulations differs for each combination of distributions for seropos/neg titres, which means that the Monte Carlo error of the estimates reported in the tables differs for each combination of distributions. Ideally, perform the stated 200 simulations for each combination. Also, as little as 12 simulations (for the normal-normal distribution combination) is too few to base inferences on. Statements like lines 225-227 in the main text (about the overrepresentation of Weibull-distributed seronegative titres among misidentified distributions) should be based on more simulations, and ideally the same number of simulations per combination of distributions.

Thank you for the useful suggestion. In the revised version of the manuscript, we have re-run the simulation study using 540 simulations, with the same number of simulated datasets for each distribution pairing (60 simulations for each of the 9 possible distribution pairings). Table S2 (previously S3) shows the number of simulations out of 540 (previously 200) where the mixture that was fitted to the simulated antibody titre dataset had the correct distribution of the seronegative and/or seropositive component (Gamma, Normal or Weibull) that the data were simulated with. The table caption has been updated to better clarify this.

Summary of changes:

- The Table caption now reads: “Number of simulations where the mixture model correctly specified the distributions of the seronegative and/or seropositive component of the simulated antibody titre datasets (Dataset C). Here, n represents the number of simulated datasets out of 540.”

Figure S1 summarises the estimated vs. true parameter values in the simulation study. Could the authors perhaps checks, and if relevant, visualise whether specific combinations of distributions where overrepresented among the outlying estimates (red bullets)?

In the revised simulation study analysis, we found 208 outliers in the estimates of muS, muI, sdS and sdI (out of 2160 (4 parameters across 540 simulations)), shown as red points in Figure S4 (Figure S1 in the original version of the manuscript). Simulations where the seronegative distribution was Weibull were overrepresented in the outliers (see revised Figure S4 below).

Summary of changes:

- S4 Figure: (**A**) True versus estimated parameter values from the mixture model fitted to the simulated datasets (Dataset C). The estimated parameters are the mean log(titre + 1) value of the seronegative/susceptible (S) and seropositive/infected (I) components ($\mu_{s}$ and $\mu_{I}$respectively) and the corresponding standard deviations ($\sigma_{s}$ and $\sigma_{I}$). Red indicates the estimates where the true parameter value was not captured by the estimates (i.e., the 95% Confidence Interval of the estimate did not contain the true value). Note that the axes limits differ for each panel. (**B**) The percentage of parameter outliers after fitting the mixture model to Dataset C, per seronegative and seropositive titre family distributions. The percentage of the total number of outliers of $\mu_{s}$, $\mu_{I}$, σS and σI (red in panel A) per distribution combination on the x-axis, where the two letters represent the seronegative (first letter) and the seropositive (second letter) distribution pair (N = normal, G = gamma and W = Weibull).

Figure S2: in the figure legend, clarify what this “%” misclassification error represents. Also, the use of linear regression to summarise the pattern is inappropriate as the “data” are bounded at zero (and the regression line dives below zero within the range of the “data”).

The y-axis of Figure S5 (Figure S2 in the original version of the manuscript) shows for each of the 540 simulated datasets, the percentage of the 3,194 titres which were misclassified as seropositive or seronegative using the optimised thresholds. The “% misclassification error” represented the proportion of misclassified titres out of the 3,194. The figure caption has been updated to better clarify this and the y-axis label has been altered. The linear regression line has been replaced with a smoothed ‘loess’ regression line.

Summary of changes:

- S5 Figure: Association between the true component mean titre values in Dataset C versus the serostatus misclassification error. The x-axis shows the difference between the true mean log(titre + 1) value of the seronegative ($\mu_{s}$) and the seropositive component ($\mu_{I}$) for each realisation over 509 simulated datasets. The titres are classified as seropositive or seronegative using realisation-specific optimised titre thresholds. The loess regression line and corresponding 95% Confidence Intervals are shown.

Figure S3: looking purely at the “data”, I would say there is no or little correlation between the two outcomes on the x and y axis. In contrast, the linear regression lines are very suggestive of a strong correlation, however, these are largely driven by very few data points towards the right end of the x axis.

Figure S6 (Figure S3 in the original version of the manuscript) has been updated after the simulation study was run with 540 new simulated datasets. After removing outliers on the far right of the x-axis, the regression lines are still suggestive of strong correlation.

Summary of changes:

-

Lines 253-255 refer to S7 Figure, but no such figure is included in the supplementary materials. Several other cited supplementary materials are not available to the reviewer.

There was a labelling issue in the Supplementary Information where previously, Figures S1-S5 in the supplementary information corresponded to Figures S5-S9 in the main text. Apologies for the confusion, we have rectified the issue now.

Lines 255-258: I’m not sure I follow why the models were fitted to the “true serostatus”, or how this was done.

This was a secondary analysis which we performed on the simulated datasets to explore whether the bias observed in the estimates from the catalytic models (which rely on serostatus classification of the titres using thresholds) was due to serostatus misclassification, or whether the bias was also present due to methodological reasons when we used the known true (simulated) proportions of seropositive subjects in the catalytic model.

Reading about the existence of multiple districts in the Vietnam data, I don’t recall reading anything about it in the methods section and whether and how this was dealt with in the modelling.

The data for Vietnam was collected in one district (Long Xuyen), and the data for Indonesia was collected in Jakarta (across 30 subdistricts). Given the small spatial scale of the range of data collection, we did not explore spatial differences. Because we do not use the subdistrict information in the Indonesian dataset, we have removed Table S1 from the Supplementary Information.

Summary of changes:

- Lines 121-125 read: “DENV IgG data from 30 urban subdistricts in Jakarta, Indonesia were collected from 3,194 children under 18 years old as part of a cross-sectional seroprevalence survey in 2014 (34) (Dataset B). Given the small spatial scale of the range of data collection, we did not account for spatial differences when modelling”

**Discussion**

For a (too) large part, the discussion section rehashes the results, with many references to tables and figures. For instance, the third paragraph starts by listing of literal estimates. As a reader, I’m left wondering what it all means. I was expecting to get at least some sense of this by the end of the first paragraph in the discussion (and then getting more details later). A more high-level description of the findings and implications would be helpful here.

Thank you for bringing this to our attention. We have changed the focus of the discussion in line with this suggestion.

Summary of changes:

- The first paragraph of the discussion now reads: “In this analysis, we explored the accuracy and bias of FOI and seroprevalence estimates obtained from mixture and catalytic models applied to serological data. The catalytic models were applied assuming a time-constant or time-varying FOI. We performed a simulation study to compare the performance of each model with known parameter values used to generate the simulated data, and observed significantly greater accuracy in FOI and seroprevalence estimates from the mixture and time-varying catalytic models than time-constant catalytic models. We observed reduced bias and uncertainty in estimates from the mixture compared to the time-varying catalytic model.”

I am missing a link to the motivation for the (a) motivation for the study as phrased in the author summary: “Characterising the transmission intensity of dengue virus in different geographic areas over time is essential to understand who is at greatest risk of infection, and to inform the implementation of interventions, such as vector control and vaccination.” If FOI estimates are important to understand where dengue transmission is most intense and to inform the implementation of interventions, bias in FOI estimates is only relevant if they muddle the relative ranking of different areas or (subsets of) populations in terms of transmission intensity and/or recency. This is not clearly discussed and I’m not sure whether the analyses and results are suitable to make statements about this.

The aim of this sentence in the author summary section was to highlight the important applications of FOI estimates , e.g. for informing policy, which is why it is important that researchers analyse potential biases in the estimates and choose the most accurate methods going forward. We agree that bias would be extremely important if it changed the relative ranking of FOI or seroprevalence estimates in different geographic areas in a country, but accurate estimates are essential for several other applications including identifying environmental drivers of transmission and assessing the suitability of the blanket rollout of interventions such as vaccination. For example, in 2016, the World Health Organisation recommended CYD-TDV dengue vaccine rollout only in places where seroprevalence was > 0.7. We have now revised the author summary to better clarify the motivation of this study, focusing on the methodological importance rather than the role of spatial heterogeneity which indeed is not the objective of the study.

Summary of changes:

- The author summary now starts with: “Characterising the transmission intensity of dengue virus is essential to inform the implementation of interventions, such as vector control and vaccination, and to better understand the environmental drivers of transmission locally and globally. It is therefore important to understand how methodological differences and model choice may influence the accuracy of estimates of transmission intensity”

**Minor comments**

- Line 52: semicolon should be a comma
- The order of the figures was mixed up in the pdf file for the reviewers, which caused some confusion at first.

Thank you for bringing these to our attention, the manuscript has been updated to account for these changes.

# Reviewer #2

This paper by Cox et al. compares different methods to estimate the force of infection and the seroprevalence of DENV from cross-sectional seroprevalence surveys. They show that serocatalytic models induce a bias in the parameter estimations compared to a mixture model. I think the authors do a good job at advocating the use of quantitative antibody titers.  I appreciated the simulation study, and the statistical analysis is done correctly.

Thank you for your feedback and positive response to the simulation study.

The authors show well the importance of using quantitative serological data to estimate key epidemiological parameters. I have however some concerns about the novelty of the work. Moreover, some of the points raised in the discussion did not convince me.

In response to the comment about the novelty of the work, we have changed the focus of our introduction and discussion to highlight the motivation and benefits of running a simulation study. Whilst several papers in recent years have used mixture models to estimate epidemiological parameters, this often is not accompanied by comparisons with catalytic models as we present here. Using a simulation study to explore the two methodologies allows an in-depth and direct comparison with known (true) values which improves our understanding of how methodological differences can affect FOI estimates, and the extent to which FOI estimates obtained using different models can be compared, which is useful for applications to real data in future work. In response to the comment about the discussion, we have revised the focus of the discussion (please see more information in the response points further in this document).

I would suggest minor changes to the figures.
-       Figure 1: the y axis need to be consistent, for instance the coverage has to go between 0 and 100. 
-       Figure 5, it would be better to have the same x axis in all panels.
-       The labels of the supplementary figures are not consistent (they start at Figure S5 in the main text, and at S1 in the supplementary material)
Reference 22: Bollaerts et al., part of the title is missing

Thank you for bringing these to our attention, the manuscript has been updated to account for these changes.

Summary of changes:

- Figure 1:

- Figure 5:

1/ Using mixture models to extract epidemiological parameters from serological studies is quite common now. The authors cite a few papers that use mixture models to estimate the FOI and claim line 97 that they are the first to use these methods to estimate DENV FOI. I’m not yet convinced that applying these methods to estimate DENV FOI is a big improvement to what was done in the various cited papers that used mixture models. Unless accounting for specificities of this virus (like primary/post-primary infections) or for instance specificities in the type of serological data, I don’t see a big novelty brought by the fact that the virus studied here is DENV. It is thus important to show more clearly the novelty of the work in the introduction.

Thank you for highlighting this issue. We have added more references to our introduction discussing further applications of mixture models to extract epidemiological parameters. We believe that a particular strength of our work is the simulation study, which allows us to conduct ‘controlled experiments’ with which we can compare the known force of infection (FOI) values used to simulate the data with the FOI estimates obtained with both mixture and catalytic models. Better understanding how methodological differences affect parameter estimates is important to assess (i) the goodness and reliability of the estimates and (ii) whether the results obtained across studies using mixture and catalytic models are comparable. The wording of our introduction has been changed to emphasise the strengths of our work and discuss in greater detail how it contributes to the growing body of evidence exploring the application of mixture models to real data.

Summary of changes:

- We have removed line 97.
- The introduction section in question now reads: “To date, mixture models have been applied to serological data to estimate the seroprevalence of infectious diseases such as parvovirus B19 and rubella in England (26,27), human papillomavirus in the Netherlands (23), measles in Italy (28), and a selection of arboviruses inlcuding DENV in Zambia (29). In addition, mixture models have been used to develop frameworks capable of distinguishing between primary and post-primary DENV infections (30,31), and recent and historical influenza A infections (32). Recently, DENV FOI was estimated using catalytic and mixture models applied to serological data collected in three locations in Vietnam (N >= 266) and in Chennai, India (N = 799) (30). In this study, the estimates from mixture models were deemed more robust than those from binary catalytic models (30). Here, we implement a simulation study to assess the ability of mixture and catalytic models to reconstruct the FOI value used for simulating the data. Furthermore, we add to the growing body of evidence exploring the use of mixture models by presenting a comparitative analysis of the DENV trasmission intensity estimates obtained from mixture and catalytic models applied to age-stratified serological datasets from Vietnam (N ≥ 2178, for years 2004-2009) and Indonesia (N = 3194, for 2014).”

2/ In the author summary, the authors write that “in high transmission areas catalytic and mixture models produced consistent estimates”. I think this is a bit misleading and hard to understand when compared to Figure 2 that show that the difference between the mixture and catalytic models increase with the FOI. The claim in the summary seems to be not general but true for the Indonesian dataset – which is obtained in a context of high circulation, and where catalytic and mixture models produced consistent estimates – but it seems that the main reason for this consistency is the little overlap in the negative and positive titer distribution.
If Figure 2 was generated with the values of the seronegative and seropositive components estimated for dataset B (the distributions and values of muS/muI), would they observe a similar difference between the mixture models and catalytic estimates? I expect no impact of the high value of the seroprevalence.

We agree that our sentence was not consistent with the results shown in the original version of Figure 2. We have re-run the simulation study as suggested by Reviewer 1. We simulated a larger number of datasets using a wider range of parameter values. The new results, which are shown in the revised version of Figure 2, show that higher FOI does not correlate with increased differences in the estimates from catalytic and mixture models. We also agree that the main reason for the consistency in the Indonesian estimates is the small overlap between the seronegative and seropositive distributions. We have updated the summary to highlight that the underlying data structure is the driving force for similarities/differences, rather than the transmission intensity (see discussion and plots in answer to 3/).

Summary of changes:

- Updated Figure 2:

3/ Line 330 : “Differences in the degree of overlap between components in real serological datasets (S8 Figure) reflect differences in transmission intensity and variable degrees of spatiotemporal heterogeneities in the risk of infection (3,37). “
The titer distributions in the datasets of Vietnam vs Indonesia are so different, that I doubt that they are simply reflecting differences in the exposure to the virus. Since it seems that different ELISA tests were used for datasets A and B, isn’t it the most likely reason for the different titer distributions?

“In datasets collected from areas which experience hyperendemic DENV circulation, one expects greater separation between the titre components because most seropositive individuals likely have had multiple infections, translating to higher antibody titres”
I don’t believe this is true. If it were true, the way I interpret the Indonesian dataset based on this claim is that some individuals have never been exposed to the virus, whereas the other ones have been exposed multiple times. Is it realistic, and is there a way to check this? I suggest to break down by age groups the titer distribution of the seropositive components. Younger kids are expected to having been fewer infections than older ones.

We believe that a combination of factors is impacting the difference in titre values between Indonesia and Vietnam, and we agree that the different ELISA tests used can be a driving factor. After performing the suggested check on the Indonesian dataset (see plots below) we see that the proportion of samples in the seropositive component increases with age however the mean value of the seropositive titres does not increase with age. We have therefore changed the wording of our discussion about the reasoning for the separation of titres in different datasets to focus on the methodological differences between datasets rather than the transmission intensity setting where the samples were collected.

Plot showing the number of titres classified as seronegative or seropositive per age in the Indonesian dataset. We see increased seropositivity in the older age groups.

Plot showing the log(titre+1) values of the seropositive individuals per age group. The red line shows the mean log(titre+1) value of the seropositive individuals. We do not see increased titre values in the older age groups.

Summary of changes:

- The paragraph originally starting at Line 330 now reads: “Differences in the degree of overlap between components in real serological datasets are likely impacted by many factors, including differences in the ELISA tests used to measure antibody titres, the age groups sampled and the underlying age structure of the population as well as the transmission setting and spatiotemporal heterogeneities in the risk of infection at the local scale. In datasets where there is clear separation in the bimodal distribution of antibody titres, catalytic and mixture models are expected to produce more similar estimates of FOI and seroprevalence as fewer samples are misclassified during the binary classification of the data needed to calibrate catalytic models (22,24). This is consistent with the results from our simulation study and with the reduced variability we observe in our FOI and seroprevalence estimates from each model when they were applied to serological data from Indonesia compared to Vietnam, where the former had higher separation of titre distributions (Figure 3, Figure 4, S3 Table).
- We have removed Figure S8
- Our concluding paragraph in the discussion now reads: “In summary, our results suggest that mixture models represent a good alternative to catalytic models to quantify DENV time-varying FOI and seroprevalence from age-stratified serological data, with potentially less bias and less uncertainty. They may be particularly useful when estimating FOI from data where there is high overlap between the component distributions, where the risk of serostatus misclassification and bias introduction when using cut-off threshold methods is greater (S5 Figure and S6 Figure).”

4/ The titer values lines 142 and in Figure 3 for dataset B are not consistent.

The label on the x-axis for Figure 3 should have read log(titre value + 1). Thank you for bringing this to our attention, the plot axis labels have been changed.

Summary of changes:

- Figure 3:

5/ lines 138-140: I’d like to see the titer t used as a threshold. For instance as a vertical line in Figure 3.

This is a great suggestion. However, for the Vietnamese dataset the ELISA tests were done in batches and there were different ‘t’ values per batch as they represented background titre values (which were accounted for when we recentred and pooled the data), so unfortunately, we cannot represent ‘t’ on the panels in Figure 3. However, for the code that we have made available on GitHub to run the simulation study, we have incorporated this suggestion for those plots (https://github.com/Tori-Cox/Mixture-catalytic-models).
