# Supplementary material for: Estimating dengue transmission intensity from serological data: A comparative analysis using mixture and catalytic models
Source: PLoS Negl Trop Dis. 2022 Jul 11;16(7):e0010592. doi: 10.1371/journal.pntd.0010592 (PMC9302823; doi:10.1371/journal.pntd.0010592)
Supplement: Attachment — Submitted filename: PNTD-D-21-01566_R1 comments LC.docx [file pntd.0010592.s010.docx]

General comments:

The authors have made considerable effort to address the reviewer comments, which is appreciated.

Methods:

Thanks for providing more explanation about what the catalytic model entails. Having a better understanding now, however, I’m somewhat puzzled by Equation 1 which describes the probability $\pi$ that someone in age group $a_{i}$ is positive, given the age-specific FOI $\lambda(a_{i})$ acting on that age group:

$$\pi\left( a_{i} \right)=1-\exp(-\lambda\left( a_{i} \right)\cdot a)$$

This equation implies the assumption that someone who is currently in age category $a_{i}$ has been exposed to an FOI of $\lambda\left( a_{i} \right)$ during his/her entire life (i.e., $a$ years), and that someone in the next age group $a_{i+1}$ has been exposed to a different FOI of $\lambda\left( a_{i+1} \right)$ for their entire life.

This doesn’t really make sense unless one assumes that these two age groups grew up in different settings. However, the introduction clearly states that the catalytic models assume that *“…rates of increase in seroprevalence between individuals who are age 𝑎 versus age 𝑎+1 are explained by high age-specific FOI (assuming the FOI is constant in time) or high time-specific FOI experienced by individuals of all ages during the period 𝑎 to 𝑎+1 years ago.”* However, Equation 1 assumes cohort-specific FOIs that are constant for the entire duration of a cohort’s existence, which is different from what the introduction states. The introduction implies that a cohort of individuals in age group $a_{i}$ have been exposed to a range of age-specific FOIs, i.e., $\lambda\left( a_{1} \right)$ during the duration of the first age catogory, then $\lambda\left( a_{2} \right)$, then $\lambda\left( a_{3} \right)$, …, through to $\lambda\left( a_{i} \right)$. An equation that satisfies the definitions in the introduction would be, assuming 1-year increments in age categories *i*:

$$\pi\left( a_{i} \right)=\pi\left( a_{i-1} \right)+\left( 1-\pi\left( a_{i-1} \right) \right)\cdot\left[ 1-\exp(-\lambda\left( a_{i} \right)) \right]$$

where one defines $a_{0}=0$ (meaning everybody is seronegative at birth) or estimates $a_{0}$ from the data (i.e., the prevalence of seropositivity in infants < 1 year of age). The first term $\pi\left( a_{i-1} \right)$ implies the assumption that individuals who are seropositive at age $a_{i-1}$ will also be seropositive at age $a_{i}$. From the authors’ reply to my previous comments, this is one of the intended assumptions. Then, the second term $\left( 1-\pi\left( a_{i-1} \right) \right)\cdot\left[ 1-\exp(-\lambda\left( a_{i} \right)) \right]$ simply implies that whoever wasn’t seropositive yet at age $\pi\left( a_{i-1} \right)$ had turned seropositive by age $a_{i+1}$ with probability $\left[ 1-\exp(-\lambda\left( a_{i} \right)) \right]$, assuming that age groups $a_{i}$ and $a_{i+1}$ differ by one year.

Discussion:

The authors make an important acknowledgement: *“However, it is important to consider the bias that will be introduced if the mixture distributions fit the titre data poorly.”* They may want to consider to discuss some potential approaches to addressing this. For instance, by implementing the mixture model in a Bayesian framework and performing posterior predictive checks on the observed vs. expected full distribution of titres.
