# Supplementary material for: Estimating dengue transmission intensity from serological data: A comparative analysis using mixture and catalytic models
Source: PLoS Negl Trop Dis. 2022 Jul 11;16(7):e0010592. doi: 10.1371/journal.pntd.0010592 (PMC9302823; doi:10.1371/journal.pntd.0010592)
Supplement: Attachment — Submitted filename: Reviewer_feedback_response_letter.docx [file pntd.0010592.s011.docx]

**Second stage reviewer response letter**

**Estimating dengue transmission intensity from serological data: a comparative analysis using mixture and catalytic models**

Victoria Cox, Megan O’Driscoll, Natsuko Imai, Ari Prayitno, Sri Rezeki Hadinegoro, Anne-Frieda Taurel, Laurent Coudeville, Ilaria Dorigatti

# Reviewer #1

Summary and General Comments
The author's efforts to address the reviewer comments are appreciated, in particular the clarification on what the models' underlying assumptions and what they are supposed to represent. This clarification has, however, raised a concern about the appropriateness of how the catalytic model was implemented in terms of maths. Equation 1 seems incorrect, or at least does not match the assumptions and model purpose described in the introduction. For details, please see the attachment.

The authors have made considerable effort to address the reviewer comments, which is appreciated.

Thank you for your invaluable feedback over the course of this review process. Please see below the response and discussion surrounding the catalytic model equations.

Methods:

Thanks for providing more explanation about what the catalytic model entails. Having a better understanding now, however, I’m somewhat puzzled by Equation 1 which describes the probability $\pi$ that someone in age group $a_{i}$ is positive, given the age-specific FOI $\lambda(a_{i})$ acting on that age group:

$$\pi\left( a_{i} \right)=1-\exp(-\lambda\left( a_{i} \right)\cdot a)$$

This equation implies the assumption that someone who is currently in age category $a_{i}$ has been exposed to an FOI of $\lambda\left( a_{i} \right)$ during his/her entire life (i.e., $a$ years), and that someone in the next age group $a_{i+1}$ has been exposed to a different FOI of $\lambda\left( a_{i+1} \right)$ for their entire life.

This doesn’t really make sense unless one assumes that these two age groups grew up in different settings. However, the introduction clearly states that the catalytic models assume that *“…rates of increase in seroprevalence between individuals who are age 𝑎 versus age 𝑎+1 are explained by high age-specific FOI (assuming the FOI is constant in time) or high time-specific FOI experienced by individuals of all ages during the period 𝑎 to 𝑎+1 years ago.”* However, Equation 1 assumes cohort-specific FOIs that are constant for the entire duration of a cohort’s existence, which is different from what the introduction states. The introduction implies that a cohort of individuals in age group $a_{i}$ have been exposed to a range of age-specific FOIs, i.e., $\lambda\left( a_{1} \right)$ during the duration of the first age catogory, then $\lambda\left( a_{2} \right)$, then $\lambda\left( a_{3} \right)$, …, through to $\lambda\left( a_{i} \right)$. An equation that satisfies the definitions in the introduction would be, assuming 1-year increments in age categories *i*:

$$\pi\left( a_{i} \right)=\pi\left( a_{i-1} \right)+\left( 1-\pi\left( a_{i-1} \right) \right)\cdot\left[ 1-\exp(-\lambda\left( a_{i} \right)) \right]$$

where one defines $a_{0}=0$ (meaning everybody is seronegative at birth) or estimates $a_{0}$ from the data (i.e., the prevalence of seropositivity in infants < 1 year of age). The first term $\pi\left( a_{i-1} \right)$ implies the assumption that individuals who are seropositive at age $a_{i-1}$ will also be seropositive at age $a_{i}$. From the authors’ reply to my previous comments, this is one of the intended assumptions. Then, the second term $\left( 1-\pi\left( a_{i-1} \right) \right)\cdot\left[ 1-\exp(-\lambda\left( a_{i} \right)) \right]$ simply implies that whoever wasn’t seropositive yet at age $\pi\left( a_{i-1} \right)$ had turned seropositive by age $a_{i+1}$ with probability $\left[ 1-\exp(-\lambda\left( a_{i} \right)) \right]$, assuming that age groups $a_{i}$ and $a_{i+1}$ differ by one year.

Thank you for bringing this to our attention and for taking the time to work out the equations above. The assumptions given in the introduction and in our reply to your previous comments are correct, and they match the methods and implementation of the catalytic model in our code (provided via GitHub). Equation 1 correctly describes the time-constant version of our catalytic model however it does not accurately describe the time-varying catalytic model we have implemented. I apologise for the confusion this has caused and I thank you again for bringing this to our attention and ensuring the correct equation was included. I have described the correct equation for our time-varying catalytic model below, which I confirm is the same model as the equation system you have suggested above, although written in a different formulation (see algebra proof below).

In addition, the term $\lambda\left( a_{i} \right)$ which was called ‘age-specific FOI’ caused confusion because it sounds like the average FOI experienced over an age group cohort’s lifetime. Instead, it refers to the FOI experienced by all age groups in the year X - $a,$ where X is the year of the serosurvey and $a$ is the age of individuals in the specific age category. The notation and description of the catalytic model have been updated to convey this, and we now discuss “year specific” FOI estimates rather than “age specific” throughout the manuscript. We believe that the updated version of the methods section is much clearer after implementing these changes.

The time-varying catalytic model equation:

$$\pi_{a,X}=1-exp\left( -\sum_{i=0}^{a} \lambda_{X-i} \right).$$

where$\pi_{a,X}$is the proportion of seropositive individuals in age group $a$, and $\lambda_{X-i}$ is the FOI in a given year $X-i$, where $X$ is the year the serosurvey was conducted.

Here, $\pi_{a,X}$ is estimated by calculating the cumulative FOI the individuals in that age group $a$have experienced over their lifetime, by summing the yearly FOI for the years in the sequence $X-a$ to $X$.

The time-constant catalytic model was described by Equation 1:

$$\pi_{a,X}=1-\exp\left( -\lambda\cdot a \right)$$

which is the same as the time-varying catalytic model equation above, given the assumption that each yearly FOI is the same (summing over the years is the same as multiplying by the age).

Comparing the time-varying catalytic model equation formulations:

Our equation:

$$\pi_{a,X}=1-exp\left( -\sum_{i=0}^{a} \lambda_{X-i} \right)$$

$$\pi_{0, X}=1-exp\left( -\lambda_{X-0} \right)$$

$$\pi_{1, X}=1-exp\left( -{(\lambda}_{X-0}+ \lambda_{X-1} \right))$$

…

…

$$\pi_{n, X}=1-exp\left( -(\lambda_{X-0}+ \lambda_{X-1}+\ldots+ \lambda_{X-n} \right)$$

Reviewer #1 equation:

$$\pi\left( a_{i} \right)=\pi\left( a_{i-1} \right)+\left( 1-\pi\left( a_{i-1} \right) \right)\cdot\left[ 1-\exp(-\lambda\left( a_{i} \right)) \right]$$

$$\pi_{0, X}=1-exp\left( -\lambda_{X-0} \right)$$

$$\pi_{1, X}=\pi_{0, X}+(1-\pi_{0, X})(1-exp\left( -\lambda_{X-1} \right))$$

$$\pi_{2, X}=\pi_{1, X}+(1-\pi_{1, X})(1-exp\left( -\lambda_{X-2} \right))$$

Substitute $\pi_{0, X}$ in $\pi_{1, X}$ equation & rearrange

$$\pi_{1, X}=1-exp\left( -\lambda_{X-0} \right)+(1-(1-exp\left( -\lambda_{X-0} \right)))(1-exp\left( -\lambda_{X-1} \right))$$

$$\pi_{1, X}=1-exp\left( -\lambda_{X-0} \right)+(exp\left( -\lambda_{X-0} \right)))(1-exp\left( -\lambda_{X-1} \right))$$

$$\pi_{1, X}=1-exp\left( -\lambda_{X-0} \right)+exp\left( -\lambda_{X-0} \right) -exp\left( -\lambda_{X-0} \right)exp\left( -\lambda_{X-1} \right)$$

$$\pi_{1, X}=1-exp\left( -\lambda_{X-0} \right)exp\left( -\lambda_{X-1} \right)$$

$$\pi_{1, X}=1-exp\left( -(\lambda_{X-0}+\lambda_{X-1}) \right)$$

Substitute $\pi_{1, X}$ in $\pi_{2, X}$ equation & rearrange

$$\pi_{2, X}=1-exp\left( -\left( \lambda_{X-0}+\lambda_{X-1} \right) \right)+(1-\left( 1-exp\left( -\left( \lambda_{X-0}+\lambda_{X-1} \right) \right) \right))(1-exp\left( -\lambda_{X-2} \right))$$

$$\pi_{2, X}=1-exp\left( -\left( \lambda_{X-0}+\lambda_{X-1} \right) \right)+\left( exp\left( -\left( \lambda_{X-0}+\lambda_{X-1} \right) \right) \right)-exp\left( -\left( \lambda_{X-0}+\lambda_{X-1} \right) \right)exp\left( -\lambda_{X-2} \right)$$

$$\pi_{2, X}=1-exp\left( -\left( \lambda_{X-0}+\lambda_{X-1} \right) \right)exp\left( -\lambda_{X-2} \right)$$

$$\pi_{2, X}=1-exp\left( -\left( \lambda_{X-0}+\lambda_{X-1}+\lambda_{X-2} \right) \right)$$

…

…

$$\pi_{n, X}=1-exp\left( -(\lambda_{X-0}+ \lambda_{X-1}+\ldots+ \lambda_{X-n} \right)$$

Summary of changes:

- Whilst updating the equations we have changed the notation from brackets to subscripts to improve readability. $\pi\left( a_{i} \right)$has been changed to $\pi_{a,X}$ and $\lambda(a_{i})$ has been changed to $\lambda_{X-i},$ which is the FOI in a given year $X-i$, where $X$ is the year the serosurvey was conducted.
- The parameter estimation section for the catalytic model, starting at line 164, now reads: “The yearly FOI, i.e., the per capita rate of infection experienced by individuals in a given year $X-i$, where $X$ is the year the serosurvey was conducted, is assumed to be either constant in time (constant across the years) or time-varying (piecewise constant across the years). When we assumed a time-varying FOI, the number of FOI estimates is equal to the number of single year age groups available in the datasets (maximum age group $A$ – minimum age group $M$). The proportion of seropositive individuals in age group $a$ during year $X$ ($\pi_{a,X}$), was estimated as in Equation 1. Here, the yearly FOI $(\lambda_{X-i})$ is summed over the lifetime of the individuals in age group $a$to give the cumulative FOI experienced by the individuals in this age group. If the minimum age group $M$ does not equal 1, then we estimated an average FOI for the $M$ years without age-specific data ($X-M$ to $X$) denoted $\lambda_{X-M}$.

$\pi_{a,X}=1-exp\left( -\sum_{i=0}^{a} \lambda_{X-i} \right).$	[Equation 1]

When we assume a time-constant FOI over the whole study period, Equation 1 can be expressed as shown in Equation 2:

$\pi_{a,X}=1-\exp\left( -\lambda\cdot a \right)$.	[Equation 2]”

- The FOI figure in the Supplementary Material (Figure S7) has been changed to represent the varying FOI estimates in terms of time rather than age on the x-axis.

Discussion:

The authors make an important acknowledgement: *“However, it is important to consider the bias that will be introduced if the mixture distributions fit the titre data poorly.”* They may want to consider to discuss some potential approaches to addressing this. For instance, by implementing the mixture model in a Bayesian framework and performing posterior predictive checks on the observed vs. expected full distribution of titres.

Thank you for this suggestion. The discussion has been expanded to cover this.

Summary of changes:

- Line 417 onwards now reads: “However, it is important to consider the bias that will be introduced if the mixture distributions fit the titre data poorly (24). In this study we accounted for this by using an information criterion to select the best fitting models from a range of options. In the future we will explore implementing the models in a Bayesian framework (22) which would allow us to perform posterior predictive checks to more robustly assess model fit. It would also be interesting to explore the FOI estimates obtained when applying a mixture model with more than two mixture distributions, which may better account for the complex immunity profiles observed in areas where multiple DENV serotypes circulate...”

# Reviewer #2

Editorial and Data Presentation Modifications?
In this revision, Cox et al. respond adequately to the requests of the reviewers and I found the article much clearer in this version.

Thank you for your invaluable feedback over the course of this review process.

1/ I have one comment about the equations in the Methods section: they are part of the text and the punctuation should therefore be made accordingly.

The methods punctuation has been changed accordingly.

Summary of changes:

- Full stops have been added to the end of each equation, aside from Equation 5 (previously Equation 4) which was part of a sentence in the text and so a comma was added.

2/ Are the datasets publicly available? This seems to be true for the Vietnam dataset and not for the Indonesian one. Please give more details about data availability.

The Indonesian and Vietnamese data are provided to us through collaboration agreements with Sanofi Pasteur and we do not have permission to share them. As part of the manuscript submission, we provided the following data availability statement which I believe will be added to the final version: “The serological data from Vietnam and Indonesia used in this work is proprietary and cannot be publicly shared. Qualified researchers may request access to the data provider. Further details on Sanofi’s data sharing criteria and process for requesting access can be found at: https://www.clinicalstudydatarequest.com. The simulated data can be recreated using code that we have made available in the GitHub repository.”
